# Little Things in Surgery

**Published:** 1888-03

**Authors:** J. E. Roach

**Affiliations:** Sipe Springs, Texas


### LITTLE THINGS IN SURGERY.

*By J. E. Roach, M. D., Sipe Springs, Texas.*

For Daniel's Texas Medical Journal.

MR. T. brought his little boy, aged five years, to my office for treatment, stating he was paralyised. History as follows: two or three weeks ago he noticed him not having good use of his legs, rather drawing than walking, and an indisposition to move; this state of affairs continued to increase until he brought him to my office, at which time he would not make any attempt to walk or use his legs. Had good use of upper extremities, no pain and slept well, no constitutional disturbance at all, to all appearances in good health; family history good, child showed to be well nurished, and general health had always been good. I examined him very closely several times and could not detect anything abnormal about the boy. I was at a loss to know what was the trouble, until I examined his penis, and I found an elongated prepuce with morbib adhesions. I informed his father that I would have to circumcise him, he consented and I placed him on the table and proceeded to operate, using Squibs chloroform. I broke up the adhesions and removed the entire prepuce, bringing the skin and mucous edges to-gether with the uninterrupted silk suture, and sent him home. I n four or five days he was brought back and he standing on his legs could run every where as good as ever.

Case 2nd. Feby., 1st, 1888. Mr. T. brought his little boy aged five and a half years, to my office for treatment, saying his boy had spells of falling to the ground, and bending his back backwards. Spells wonld come on once or twice a day, and was gaining in freqnency and intensity. Every other way he was all right, no fever or constitutional disturbance at all; he had been having these paroxysms for five or six weeks; his family history was good. I at once examined his penis and found an elongated prepuce, and proceeded to operate (circumcise) as in case No. 1. Four days after he was brought back to my office and had some of the sutures removed, his father stating that his boy was well and no more spells after operating. Now brethren of the profession, I only report the above cases because we are liable to overlook these little things in our practice, or in other words they are calculated to throw us off the track. I must say in conclusion, examine the little fellows' penis, and if you are in doubt proceed to operate; remove the prepuce and give your little patient the benefit of an early operation.

